# Complete Genome Sequence of Hospital-Acquired Methicillin-Resistant Staphylococcus aureus Strain WCUH29

**DOI:** 10.1128/MRA.00551-19

**Published:** 2019-06-06

**Authors:** Ting Lei, Ying Zhang, Junshu Yang, Kevin Silverstein, Yinduo Ji

**Affiliations:** a Department of Veterinary Biomedical Science, College of Veterinary Medicine, University of Minnesota, St. Paul, Minnesota, USA; b Minnesota Supercomputing Institute, University of Minnesota, Minneapolis, Minnesota, USA; University of Rochester School of Medicine and Dentistry

## Abstract

The hospital-acquired methicillin-resistant Staphylococcus aureus (HA-MRSA) strain WCUH29 has been intensively and widely used as a model system for identification and evaluation of novel antibacterial targets and pathogenicity. In this announcement, we report the complete genome sequence of HA-MRSA WCUH29 (NCIMB 40771).

## ANNOUNCEMENT

Staphylococcus aureus is an important pathogen that can cause a variety of diseases worldwide. The continuing emergence of methicillin-resistant S. aureus (MRSA) associated with both hospital-acquired and community-acquired infections has resulted in serious health care problems due to the limited effective treatment options (1, 2).

The S. aureus WCUH29 strain is a human clinical isolate from the Children’s University Hospital in Warsaw, Poland (3). WCUH29 is a MRSA strain that is resistant to multiple antibiotics, including methicillin, amoxicillin, and kanamycin (4, 5). This organism has been successfully used to investigate the pathogenesis of S. aureus in animal models of infection, including a murine hematogenous pyelonephritis infection model (3, 6, 7), and an intraperitoneal infection that can cause mortality to infected mice (7, 8). This strain has been intensively utilized for validation of antibacterial agents (7, 9, 10) and potential target genes for the development of novel antibacterial agents (11). We used this strain as a model system for comprehensive identification of genes essential for bacterial survival (12). In this study, we determined the complete genome sequence of WCUH29, which enables us to further perform comparative genomics studies and determine the impact of genetic background on a given gene’s function.

A single colony of WCUH29 was picked up from a sheep blood agar plate, inoculated into tryptic soy broth, and incubated at 37°C with shaking (225 rpm). Genomic DNA was purified from the bacterial cells of stationary-phase culture using a genomic DNA purification kit (Promega, Wisconsin WI). PicoGreen DNA quantitation, library construction with a TruSeq Nano DNA sample preparation kit (Illumina), and DNA sequencing were performed at the University of Minnesota Genomic Center (UMGC). One hundred paired-end cycles of DNA sequencing were conducted using a HiSeq 2500 platform (Illumina). The sequence data were transferred to the Minnesota Supercomputing Institute (MSI) for storage.

The genome was sequenced using a PacBio RS II system on two single-molecule real-time (SMRT) cells, which generated 184,878 filtered reads with a mean length of 6,255 bp. *De novo* assembly was performed using the Hierarchical Genome Assembly Process (HGAP) version 3 (13) with the settings filter out subreads shorter than 5,000 and set the minimum seed read to 16,000, the target coverage to 30, and the genome size to 2.9 Mb, which produced one assembled sequence (2,909,904 bp). Independent Illumina sequencing of the same strain produced 9,985,766 100-bp-long paired DNA reads. Pilon version 1.10 (14) was used to polish the PacBio assembly using this set of short DNA reads. It corrected 1 single base and inserted 29 1-bp indels genome-wide. By comparing the sequencing to 48 known S. aureus genomes, such as those of N315, MW2, Mu3, Mu50, COL, and MSSA476, we determined the origin of replication and reorganized the genome sequence to start from the origin of replication. Since we did not identify overlapping sequences, we used an “N” to fill the junction between the original start and end positions. The final assembly harbors a circular chromosome of 2,909,904 bp with a G+C content of 32.9%.

The Rapid Annotation Transfer Tool (RATT; time-stamp, 14 December 2011) (15) was used to annotate the assembled WCUH29 genome via comparisons with those of 4 known S. aureus strains (GenBank accession numbers AP009324, BA000017, BA000018, and CP001844). Rapid Annotations using Subsystems Technology (RAST) (myRAST version 36) (16) was also used to annotate the genome in order to predict WCUH29-specific genes. The final annotation of WCUH29 includes 3,004 coding genes, 35 rRNA genes, 124 tRNA genes, 2 transfer-messenger RNA (tmRNA) genes, and 2 pathogenic islands.

### Data availability.

The genome sequence of Staphylococcus aureus WCUH29 has been deposited in NCBI GenBank under the accession number CP039156 and BioProject number PRJNA531521.
